# Systematic Development of the ReWin Application: A Digital Therapeutic Rehabilitation Innovation for People With Stroke-related Disabilities in India

**DOI:** 10.2196/40374

**Published:** 2022-11-24

**Authors:** Sureshkumar Kamalakannan, Vijay Karunakaran, Ashwin Balaji Kaliappan, Ramakumar Nagarajan

**Affiliations:** 1 Department of Social Work Education and Community Wellbeing Faculty of Health and Life Sciences Northumbria University Newcastle Upon Tyne United Kingdom; 2 South Asia Centre for Disability Inclusive Development and Research Indian Institute of Public Health Hyderabad Public Health Foundation of India Hyderabad India; 3 Department of mHealth Design and Development InGage Technologies Pvt, Ltd Chennai India; 4 Neurological Rehabilitation Department Chennai Advanced Rehabilitation Centre Chennai India

**Keywords:** stroke, telerehabilitation, neurological rehabilitation, disability, India, rehabilitation, recovery, stroke care, patient care, digital technology, feasibility, acceptability, digital therapy

## Abstract

This is a viewpoint paper that aims to describe the systematic approach to the development of a technology-driven stroke rehabilitation innovation to manage disabilities following a stroke at home in India. This paper intends to sensitize public health innovators and intervention development experts about the important aspects that need to be considered to develop a culturally sensitive, patient-centered, scalable solution for stroke care using technology. Stroke has been the second-leading cause of death and the third-leading cause of disability globally for the past 3 decades. The emerging technological innovations for stroke care were predominantly designed and developed by digital technology experts as stand-alone products with very minimal efforts to explore their feasibility, acceptability, and, more importantly, scalability. Hence, a digital therapeutic rehabilitation innovation for people with stroke-related disabilities in India was systematically developed and is being evaluated. ReWin is an innovation that is technologically driven and envisions digital therapeutics as a medium for the provision of rehabilitation to persons with disabilities. It is conceptualized and developed based on the International Classification of Functioning, Disability and Health. ReWin encompasses specific technological aspects to enable its scientific framework and conceptualization to suit the context and needs of stroke care providers and consumers. The framework is built with 2 separate applications, one for the providers and one for the patients and caregivers. Each of these applications has a specific inbuilt design to add data about the demographic details of the user, stroke severity using the National Institute of Health Stroke Scale, and self-assessment of disability measured by the modified Barthel Index. Users can communicate with each other and decide on their therapeutic goals, therapy training information, and progress remotely from where they are. The ultimate outcome expected from the ReWin innovation is a continuum of care for stroke survivors that is effective, safe, and of good quality. Systematic development cannot make the intervention scalable. The intervention needs to be evaluated for its feasibility, acceptability, and effectiveness. Currently, ReWin is being evaluated for its feasibility and acceptability. The evaluation of ReWin will provide an opportunity to develop a scalable solution for empowering therapists and persons with disabilities, in general, to objectively self-manage their treatment. Findings from this study will also provide valuable information about the resources required to deliver such interventions in resource-constrained settings like India.

## Introduction

Stroke has been the second-leading cause of death and the third-leading cause of disability globally for the past 3 decades [1]. There have been several innovations to meet the growing need for stroke rehabilitation in the community [2]. Most recently, the approach to innovations for stroke rehabilitation has amalgamated the strengths of technology and digital therapeutics [3]. However, these technological innovations for stroke care were predominantly designed and developed by digital technology experts as stand-alone products with very minimal efforts to explore their feasibility, acceptability, and more importantly, scalability [4]. Perhaps this could be one reason why these technologically driven rehabilitation innovations have not been optimally used in primary stroke care, especially in the context of low- and middle-income countries including India.

As recommended by the Medical Research Council, United Kingdom, the development of innovative interventions, especially those that are complex, must be systematic and phased [5]. This will enable the design as well as the development of context-specific, culturally sensitive, and patient-centered interventions [6]. It is also important that these technological innovations connect patients with providers of rehabilitation. Most of the innovations for stroke rehabilitation that are available in the market are aimed at supporting stroke survivors or stroke service providers [7]. This potentially creates a gap in the continuum of care between the users and providers of stroke care, and therefore, the supply of rehabilitation services could never meet the demands [8]. The development of innovative stroke rehabilitation interventions that consider the aspects of feasibility, acceptability, and scalability is therefore of utmost public health importance [9]. It also stresses the importance of innovations targeting the continuum of care, especially for a condition like a stroke, which results in a long-term permanent disability [10].

In this paper, we aim to describe the systematic approach to the development of a technology-driven stroke rehabilitation innovation to manage disabilities following a stroke at home in India. The innovation is called ReWin. ReWin is a digital therapeutics platform conceptualized, developed, and owned by TNQ InGage Technologies, a company based out of Chennai, India. It is an innovation that was systematically designed with the utmost consideration for scalability and a continuum of care. This paper intends to sensitize public health innovators and intervention development experts about the important aspects that the authors considered to develop the ReWin innovation, which is a culturally sensitive, patient-centered, scalable solution for stroke care using technology in India. The paper highlights the technical as well as scientific aspects of the ReWin innovation and its implications for addressing the growing burden of stroke and the demand for stroke care in India as well as in similar contexts.

### ReWin Conceptualization

**Figure 1:**
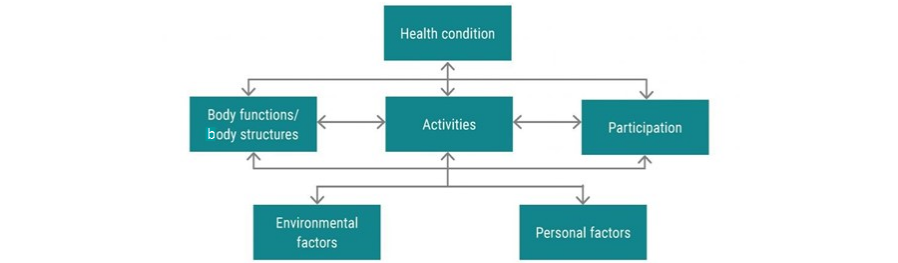
Bio-psycho-social model of the International Classification of Functioning, Disability and Health.

ReWin is a technology-driven innovation for stroke care that aims to provide a scalable solution for stroke care and subsequently generate evidence for informed decision-making among both the consumers and providers of stroke rehabilitation and care. It is conceptualized as an innovation that could visualize disability through a bio-psycho-social lens as defined by the International Classification of Functioning, Disability and Health [11]. This conceptualization enables ReWin to move beyond the boundaries of impairment-based rehabilitation, which is a purely medical model, to an inclusive model that considers activity limitations and participation restrictions, including the environment in which one experiences stroke-related disabilities. Figure 1 describes this conceptualization.

Although it looks straightforward, it is not easy to translate this conceptualized innovation into a scalable solution, especially in a country such as India where there is no general system for the rehabilitation of persons with disabilities [12]. Even the national program for noncommunicable diseases, which includes stroke, does not have an operational strategy to address the unmet need for disability and rehabilitation among stroke survivors [13]. In addition, rehabilitation services for stroke survivors, especially outside the hospital setting, are hardly available in India. This provides the opportunity to tap the strengths of technology and optimize it for bridging the existing gaps in the provision of rehabilitation for stroke survivors and meeting their needs, especially outside the hospital setting.

### Scientific Framework of ReWin

ReWin’s scientific framework is more aligned with its conceptualization. Rehabilitation services for people with disabilities in general have been inaccessible in the Indian context until now [14]. Whatever is available and accessible is limited to the major cities and provided predominately by private service providers. More importantly, rehabilitation services are restricted only to hospital or institutional settings [15]. Services for people with disabilities outside the hospital context are hardly nonexistent, and this provides an opportunity as well as implies a need for innovation to address these gaps.

**Figure 2:**
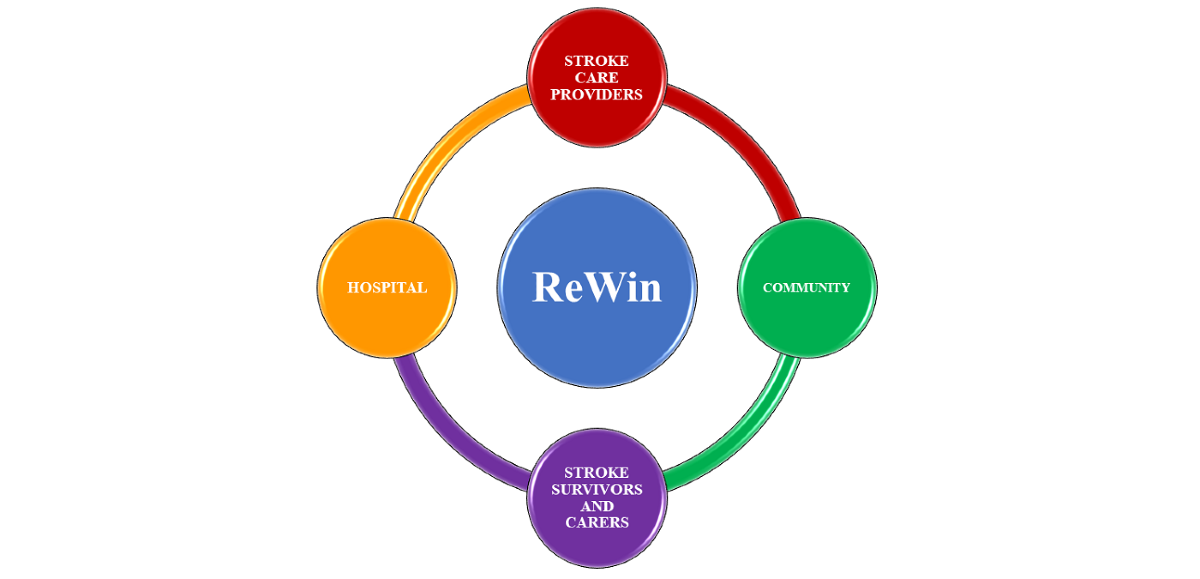
Continuum of care framework of ReWin.

The key aspects of ReWin’s framework are the continuum of care as well as the bio-psycho-social model for disability conceptualization, which is nonexistent for people with disabilities in general in an Indian context. Communication of the therapy needs and rehabilitation plans between therapists and stroke survivors and their families is crucial to achieving a continuum of care. ReWin ensures the follow-up of stroke survivors post discharge from their hospital. The application also envisions follow-up for functional independence, enabling stroke survivors to decide what goals need to be achieved based on their self-assessment of activities of daily living (ADL). Lastly, the scientific framework is not just educational. It enables supervised therapeutic rehabilitation at home using video-based educational content as well as the sensor- and virtual reality–based therapeutic training supervised remotely by the therapists based at the hospitals or rehabilitation centers. Figure 2 depicts the continuum of care framework of ReWin.

### The Technological Framework of ReWin

ReWin encompasses specific technological aspects to enable its scientific framework and conceptualization to suit the context and needs of stroke care providers and consumers. The framework is built with 2 separate applications, one for the providers and one for the patients and caregivers. Each of these applications has a specific inbuilt design to add data about the demographic details of the user, stroke severity using the National Institute of Health Stroke Scale (NIHSS), and self-assessment of disability measured by the modified Barthel Index. Users can communicate with each other and decide on their therapeutic goals, therapy training information, and progress remotely from where they are.

### ReWin Stroke Rehabilitation Intervention

The ReWin stroke telerehabilitation intervention is designed to provide continuum care for stroke survivors in India. It is designed and developed to address the unmet need for stroke rehabilitation in the country by bridging the gap between stroke rehabilitation clinicians or health professionals and stroke survivors. The stroke experts considered for delivering the intervention are stroke physicians, neurologists, physiotherapists, occupational therapists, rehabilitation nurses, and other rehabilitation experts. The stroke survivors considered for using this innovation are those who get treated in a hospital facility for their acute stroke and are discharged home after being medically stabilized for their acute stroke at the hospital. The intervention consists of two key components: (1) ReWin Stroke Survivor app and (2) ReWin Therapist app. Both apps will have the capability to communicate with each other.

### ReWin Stroke Survivor App

**Figure 3:**
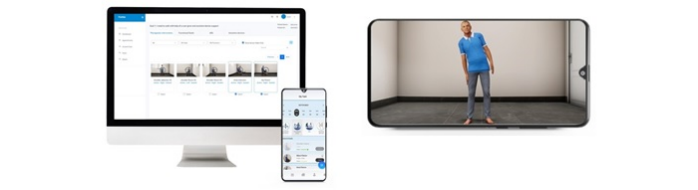
Mobile-based therapeutic videos.

**Figure 4:**
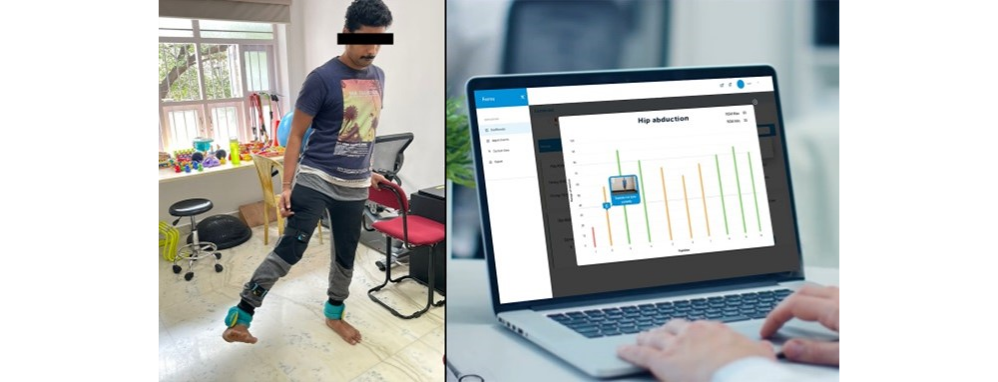
Wireless sensor for active, active-assisted, and passive therapeutic exercise.

**Figure 5:**
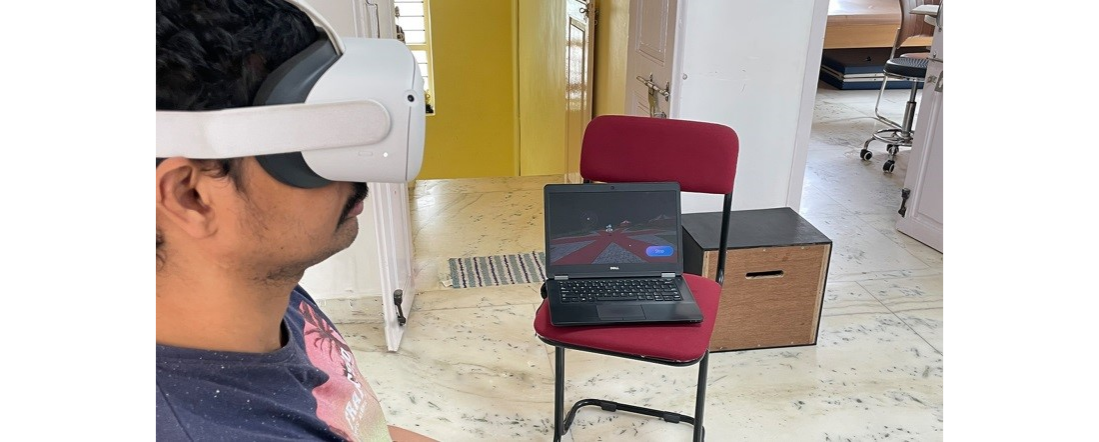
Headset of the head-mounted display for stroke rehabilitation.

This is a patient-specific app that would provide educational information and guide stroke survivors to understand stroke and their functional problems, seek uninterrupted, organized stroke care from stroke experts based at the hospital, and set realistic goals that could help them meet their poststroke rehabilitation needs based at home. The intervention includes the following components:

1. Information on stroke: a video-based education for caregivers and stroke survivors on stroke such as what is stroke, how a stroke occurs, common symptoms of stroke, and warning signs of stroke in regional languages.
2. Self-care assessment: a self-care assessment is designed to assess and evaluate the participation of stroke survivors in basic ADL using a standardized assessment of ADL. All the questions are developed using the modified Barthel index assessment for ADL as the core logic. This assessment is completed by the stroke survivors or their caregivers themselves.
3. Functional goal-setting: the patient app will suggest functional goals to the patient based on the results of the self-care assessment. Patients can select short-term functional goals in areas where they want to see improvement in their ADL.
4. Therapeutic expert consultation: these goals will be shared digitally with the stroke experts using a single point-of-access triage facilitator in the hospital for further rehabilitation and care planning (web-based consultations, goal planning, and follow-up) for the stroke survivors based at their homes.
5. Home-based self-management or caregiver-supported management of the physical disability: this is the core therapeutic component of the ReWin innovation. It consists of 4 critical domains for stroke rehabilitation. They are (1) home-based therapeutic exercises, (2) functional skills or preparation for daily living, (3) ADL, and (4) assistive devices. The content of all these domains is stored in the server of ReWin. This content can be reviewed and recommended to stroke survivors by the therapists through their app. Following this, the recommendations will be automatically available for the users through their patient app.
  - Home-based therapeutic exercises: in this section, self or caregiver-mediated home-based training with support using a customized exercise program as prescribed by their expert therapist can be performed. Stroke survivors and caregivers would be asked to follow the exercises provided in the app based on expert guidance. Home-based therapeutic exercises will be provided on 3 platforms. Each platform has its uniqueness.
    - Guided 3D video-based therapeutic exercises: in this platform, ReWin offers 3D animated videos that will guide and train stroke survivors and caregivers on how to do their daily therapeutic exercises as prescribed by their expert therapist. The therapeutic exercise library will have a wide selection of passive, active-assisted, and active exercises (Figure 3).
    - Wireless bio-feedback sensors: bio-feedback sensors combined with a mobile app will help to coach, track, and remotely monitor patients to enhance and improve their rehab experience. These sensors are nonhazardous and can track the range of motion, time taken to complete a task, and movement smoothness. All these data will be monitored and shown in the expert app dashboard. A wide range of passive, active-assisted, and active exercises will be available on this platform (Figure 4).
    - VR therapeutic games: innovative, immersive, therapeutic applications that address a wide variety of active range of motion exercises focused on ADL through virtual reality games. Each game is uniquely designed and curated for a particular active range of motion exercise. The purpose of this intervention is to provide immersive, engaging, safe, and higher dosage interventions in a home or clinical setting for patients with stroke (Figure 5).
  - Functional skills or preparation for daily living: a virtual reality–based education of stroke survivors and caregivers on ways to perform their functional activities, such as positioning themselves in bed and chair, bed mobility, mobilizing in bed, chair, and wheelchair, and transfers, will be available for viewing and performing under expert guidance.
  - ADL: a virtual reality–based education of stroke survivors and caregivers on ways to perform their functional activities such as brushing, grooming, bathing, dressing, and walking will be available for viewing and performing under expert guidance.
  - Assistive devices: a virtual reality–based education of stroke survivors and caregivers on assistive devices that can help with their ADL, details of the device, how to wear them, and perform their day-to-day activities is provided.

### ReWin Stroke Expert App

The ReWin stroke expert app is specifically designed to enable stroke care experts in a hospital facility to ensure a continuum of care by engaging with stroke survivors who are discharged from the hospital and are living at home digitally in real-time. This app is designed to serve the following purposes:

1. Web-based consulting: this app enables stroke rehabilitation experts to consult with a stroke survivor virtually rather than consulting at the hospital. The experts can provide appointments for specific time durations and could organize care for their patients who have already been discharged and keep track of them.
2. Stroke severity assessment: the app has an inbuilt NIHSS. This standardized assessment will enable expert clinicians to assess and evaluate the stroke severity, and disabilities or impairments due to stroke. The assessment consists of structured questions to evaluate physical, visual, cognitive, speech, and sensory deficits and is modeled on the NIHSS. The outcome of the assessment will reflect the severity of the stroke. This assessment is done face-to-face with the present pilot.
3. Digital goal-setting: stroke expert clinicians using this app can view the goals set by their patients and can consult to set realistic goals together with the patient and keep track of them. This could serve the purpose of both achieving it with optimum resources and ensuring the prognosis is documented and patient progress is objectively guided.
4. Web-based supervision of therapy: based on the goals set for a particular patient, the therapist can set the therapeutic interventions mentioned in the survivor’s app, such as therapeutic exercises, functional skills, ADL, and assistive devices, as per the needs of the patient and ask their patients to understand how it is done and practice it at home under their supervision. Under the therapeutic exercises section of the app, the therapist can select the exercises and repetitions or dosages on a week or fortnight basis for their patients and track real-time progress objectively. Our platform also has the capability to recommend exercises as per the patient’s goals, but the final decision will be taken by the concerned expert clinician.
5. Web-based follow-up: similar to consultation and supervision, the app will enable expert clinicians to conduct web-based follow-ups of their patients who have been discharged from hospital-based rehabilitation and care but who have been using the patient app for this intervention and are being cared for by the expert clinicians irrespective of the patient’s living location globally.

### ReWin Stroke Telerehabilitation App Technological Flow

**Figure 6:**
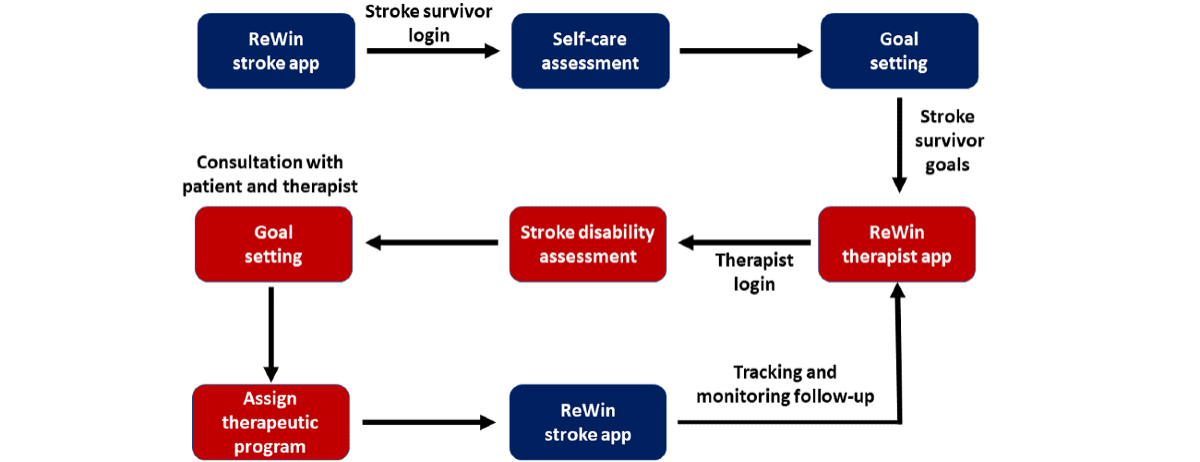
The operational flow of the intervention in terms of synchronized patient and clinician applications.

The ReWin stroke survivor app and the therapist app are designed to communicate with each other to provide continuous support to the therapist and stroke survivor. A summary of the workings of the app is detailed below and in Figure 6.

- A stroke survivor logs into the app and performs a self-care assessment. Goals are suggested to the patients based on their assessment outcomes.
- Stroke survivors and caregivers select goals that they want to see improved in their daily lives.
- The goal outcome is communicated to a therapist.
- The therapist performs a stroke severity and disability assessment on the stroke survivor, and the therapist, stroke survivors, and caregivers consult and finalize their goals for improvement.
- The therapist prescribes intervention based on the modified goals on a weekly or fortnightly basis.
- Therapeutic intervention is sent to the ReWin stroke survivor app for daily usage.
- Stroke survivors and therapists communicate digitally using the app at their discretion to achieve therapeutic goals and experience a continuum of care.

## Discussion

### Principal Findings

Globally, telerehabilitation has been widely accepted and promoted in stroke care [16]. Several innovations have been developed worldwide and tested for their feasibility and effectiveness [17]. Telerehabilitation for stroke includes a wide range of innovations targeting the diverse aspects of stroke care, ranging from lifestyle coaching to reducing various types of impairments as well as functional rehabilitation [18]. It is evident that telerehabilitation for stroke care can be a very useful adjunct to conventional stroke care at rehabilitation centers and hospitals and in situations where there is no other option [18]. There is also evidence to suggest that telerehabilitation interventions have either better or equal salutary effects on physical, psychological, and cognitive impairments compared with conventional face-to-face therapy [17]. Although there is evidence for the feasibility of such innovations, the strategy used in these innovations to promote a continuum of care is still unclear [16-19]. Technological innovations for stroke care targeting the posthospital discharge period have not been shown to reduce impairments, improve independence in ADL, or improve quality of life [16]. This is especially true because telerehabilitation innovations targeting continuum care are hardly developed and evaluated, particularly in low- and middle-income countries such as India [20].

ReWin innovation targets a continuum of care for stroke survivors that is effective, safe, and of good quality. Patients who seek treatment for stroke in a hospital get comprehensive treatment for acute stroke, and they get discharged home as soon as they are medically stable [21]. Poststroke disability is poorly understood and managed because we do not have a continuum of care outside the hospital setting in a country such as India [22]. This is true even from the government’s health care perspective. The ReWin innovation bridges this important gap.

First, it enables the continuum of care for stroke survivors after hospital discharge at their home (patient app). Second, it enables the hospitals to organize their services systematically in the community for their patients and strengthen their service provision outside the hospital and have a follow-up of their patients even after they leave the hospital facility through this innovative technology-driven intervention. Systematic development cannot make the intervention scalable [8]. The intervention needs to be evaluated for its feasibility, acceptability, and effectiveness [23-25].

The key limitation of this viewpoint paper is that it does not provide the results of the feasibility assessment with sufficient data in detail. This is especially because the pilot study is currently in progress. However, once it is complete, we will ensure we report the results of the evaluation as a separate publication. Currently, ReWin is being evaluated for its feasibility and acceptability. It will be subsequently evaluated for its effectiveness too. Given our experience in developing the ReWin intervention, we foresee that affordability (costs) as well as the digital literacy of the users, particularly those who are older adults, will be key barriers to implementation that must be evaluated. The evaluation of ReWin will provide an opportunity to develop a scalable solution for empowering therapists and persons with disabilities, in general, to objectively self-manage their treatment. Findings from this study will also provide valuable information about the resources required to deliver such interventions in resource-constrained settings such as India.

### Conclusions

Technology-based innovations for stroke care are absolutely essential to bridge the gaps in access to rehabilitation and to enhance recovery following stroke, particularly after hospital discharge. Innovations such as the ReWin are warranted for development as well as systematic evaluation. The innovation must connect stroke care providers in hospitals and rehabilitation centers to stroke survivors and their families in the community. The development of innovations without a strategy to ensure a continuum of care can only be efficacious in the ideal environment and might not be a scalable solution to ensure effective community-based stroke care, particularly in a country such as India.

## Abbreviations

ADL: activities of daily living
NIHSS: National Institute of Health Stroke Scale
